# Applications and therapeutic mechanisms of action of mesenchymal stem cells in radiation-induced lung injury

**DOI:** 10.1186/s13287-021-02279-9

**Published:** 2021-03-25

**Authors:** Shiying Niu, Yueying Zhang

**Affiliations:** 1 Institute of Basic Medicine, The First Affiliated Hospital of Shandong First Medical University, Jinan, 250062 Shandong China; 2 Department of Experimental Pathology, Institute of Basic Medicine, Shandong First Medical University (Shandong Academy of Medical Sciences), Jinan, 250062 Shandong China

**Keywords:** Mesenchymal stem cell, Radiation-induced lung injury, Stem cell therapy

## Abstract

Radiation-induced lung injury (RILI) is one of the most common complications associated with radiotherapy, characterized by early-stage radiation pneumonia and subsequent radiation pulmonary fibrosis. However, effective therapeutic strategies for RILI are currently lacking. Recently, an increasing number of studies reported that mesenchymal stem cells (MSCs) can enhance the regeneration of damaged tissue, modulate the inflammatory response, reduce the levels of fibrotic cytokines and reactive oxygen species, and inhibit epithelial-mesenchymal transformation. Interestingly, MSCs can also exert immunosuppressive effects, which highlights a new potential therapeutic activity of MSCs for managing RILI. Here, we reviewed the potential applications and therapeutic mechanisms of action of MSCs in RILI, which will represent a good compendium of information for researchers in this field.

## Background

Radiation therapy is one of the most significant treatment modalities for multiple thorax-associated neoplasms, such as lung cancer [1]. Radiation-induced lung injury (RILI) is a common complication of radiotherapy and limits the therapeutic dose of radiation that can be administered to effectively control the tumors. More than 40% of patients treated with high-dose radiotherapy are diagnosed with RILI [2]. Owing to the complexity of its pathology, the molecular mechanisms underlying the development of RILI remain poorly understood. Indeed, RILI is still a serious and complicated lung disease because of the absence of specific treatments [1, 3]. Recently, some studies suggested MSC-based therapy as a promising and potential treatment for RILI [3, 4]. MSCs is a population of multipotent cells that can enhance the regeneration of damaged tissues, modulate the inflammatory response, reduce the levels of fibrotic cytokines and reactive oxygen species (ROS), and inhibit epithelial-mesenchymal transformation (EMT). Moreover, several studies reported the effects of MSCs on chronic lung diseases [3]. Therefore, interest in the therapeutic potential of MSCs has increased significantly and has been used for treating radiation-associated diseases [3, 5]. Here, we have reviewed the recent progress regarding potential applications and on the understanding of the therapeutic mechanisms of MSCs in RILI. We focused on the immunosuppressive effects of MSCs, which may represent an important therapeutic advantage of MSCs to treat RILI and pave its way from the bench into the clinic.

## Pathogenesis of RILI

**Fig. 1:**
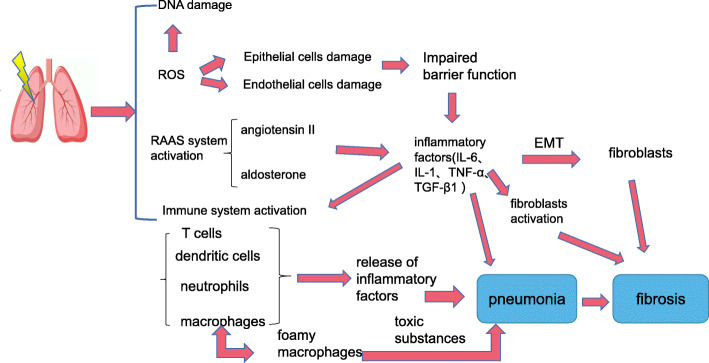
The primary mechanisms /pathogenesis of radiation-induced lung injury (RILI): direct DNA damage; the generation of ROS; RAAS system activation; the activation of the immune system. ROS, reactive oxygen species; RAAS system, renin-angiotensin-aldosterone system; EMT, epithelial-to-mesenchymal transition

Radiotherapy is an effective strategy to treat cancer; however, radiation can damage the pulmonary epithelial and endothelial cells, resulting in RILI (Fig. 1). RILI is a complicated pathological process, including early radiation pneumonia (RP) and late radiation pulmonary fibrosis (RILF). RP usually develops few hours after the radiation is administrated and lasts for 1–2 months, while RILF onset occurs after 2 months, which generally is indicative of irreversible damage. The precise molecular mechanisms underlying RILI are still unclear [5]. Direct DNA damage, generation of ROS, and activation of the immune system are the primary mechanisms [6].

Few minutes after lung irradiation, damage to DNA and organelles triggers intracellular signal transduction that results in gene expression changes. In addition, the ionization of water molecules generates ROS, which will in turn promote genomic and mitochondrial DNA ROS, and consequent inflammatory and immune responses [7, 8]. Furthermore, ROS can cause apoptosis of alveolar epithelial cells (AECs) and endothelial cells, thereby destroying the epithelial-endothelial barrier function and vessel integrity [6]. Large amounts of cytokines are released from injured lung tissue that will recruit inflammatory cells into the alveolar cavity to further expand the inflammatory responses [3, 5, 9]. Fibroblasts are also activated to further differentiate into myofibroblasts, which will promote lung fibrosis [10].

More importantly, radiation can also activate the immune system, such as monocytes, T cells, dendritic cells (DCs), and neutrophils [3]. The immune cells will produce pro-inflammatory cytokines that will further activate fibroblasts and the inflammatory responses. Macrophages, which are a type of monocytes, play important roles in the activation of the immune system. Studies have shown that the persistent activation of macrophages can affect tissue repair [11]. Lipid metabolism is also affected by radiation, of which oxidation byproducts will promote the formation of foamy macrophages [12], which are formed when these cells absorb excessive lipoproteins. In turn, foamy macrophages can support apoptosis or necrosis and release toxic substances that promote inflammation. Six months after radiation, activated macrophages not only produce a large number of pro-inflammatory cytokines, but also increase oxygen consumption, leading to the formation of a hypoxic environment that stimulates the production of ROS and pro-fibrotic factors, aggravating tissue damage [11, 13]. T helper cells type 1 and 2 play important roles in RILI. Th1 cells secrete interferon-γ (IFN-γ), an important anti-fibrotic factor, which when increased can lead to RP [14]. In turn, Th2 cells secrete interleukin (IL)-4 and IL-13 in RILI [15]. Indeed, IL-4 levels were found to be significantly increased in the serum of patients with idiopathic pulmonary fibrosis and RILI [16, 17], with the expression of collagen and fibronectin being significantly increased in fibroblasts treated with IL-4 in vitro [18]. In addition, IL-13, an important fibrotic cytokine, is closely related to liver fibrosis and lung fibrosis [19]. Chung et al. found that IL-13 expression increased significantly in RILI, whereas the pathological changes were significantly improved in IL-13-knockout mice with RILI [20]. Activated fibroblasts secrete a large amount of prostaglandin E2 (PGE2), which can inhibit T cell differentiation into Th2 cells. However, PGE2 production decreases in the later stage of RILI owing to increased fibroblast differentiation and epithelial cell damage, which will support the development of fibrosis [14, 15].

Changes in the renin-angiotensin-aldosterone system (RAAS) may also play a crucial role in the development of RP [21]. Angiotensin II is a pro-inflammatory factor contributing to the development of the inflammatory response. Rosenkranz et al. showed that angiotensin II levels increased in the early phase post-radiation [21], thereby stimulating the secretion and activation of the transforming growth factor (TGF)-β1, leading to lung damage. Several studies have shown that aldosterone can also damage various organs. Under pathological conditions, aldosterone-induced TGF-β1 expression can promote fibrosis [22]. In addition, Zhao et al. pointed out that aldosterone was involved in angiotensin-induced cardiac injury. Therefore, the RAAS may participate in RILI [23].

The role of cytokines in RILI should also be considered. Radiation also triggers the release of several signaling factors, such as IL-1, IL-6, tumor necrosis factor (TNF)-α, and TGF-β1 [3, 9]. Among these, TGF-β1 is the most crucial factor in RILI. TGF-β1 can promote the differentiation of fibroblasts into myofibroblasts and it can also induce EMT, which will actively support the development of RILF [6, 24]. After lung injury, damaged epithelial cells and inflammatory cells can also release the matrix metalloproteinases (MMP) 2 and 9 to activate TGF-β1 secretion. However, whether MMP2/9 induces EMT in AECs remains unknown [25].

### Characteristics of MSCs

**Fig. 2:**
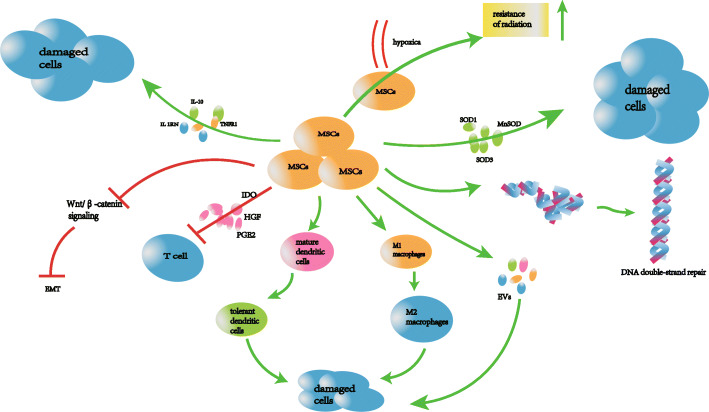
Molecular mechanisms of MSC-based therapy for RILI through the following aspects: reduction of inflammatory and fibrosis reaction; resistance of oxidative stress; inhibition of EMT; immunosuppressive properties; release of extracellular vesicles. EMT, epithelial-to-mesenchymal transition; IL-1β, interleukin 1β; IL-6, interleukin 6; IL-10, interleukin 10; TNF-α, tumor necrosis factor α; SOD1, superoxide dismutase 1; SOD3, superoxide dismutase 3; MnSOD, manganese superoxide dismutase; HGF, hematopoietic growth factor; EVs, extracellular vesicles; PGE2, prostaglandin E2; IDO, indoleamine 2,3-dioxygenase

Among stem cells, MSCs possess considerable therapeutic potential for regenerative medicine and have been extensively explored [9, 26]. MSCs are ubiquitous, but it can be mainly found in the bone marrow, umbilical cord, and adipose tissue. They have low immunogenicity and are capable of differentiation and proliferation. Furthermore, they can modulate the inflammatory responses and promote repair of damaged tissue, thereby showing considerable potential for RILI treatment [3, 5, 9, 26]. As shown in Fig. 2, multipotent MSCs can enhance regeneration of damaged tissue by modulating the inflammatory response, reducing the levels of fibrotic cytokines and ROS, and inhibit EMT.

### Recent advances in molecular mechanisms of MSC-based therapy for RILI

#### Reduction in inflammatory and fibrotic cytokine levels

As a pulmonary complication, radiation can induce the release of various pro-inflammatory cytokines, such as IL-6, IL-1β, and TGF-β1, which can induce the activation of fibroblasts and consequent development of fibrosis [27, 28]. However, MSCs can reduce inflammation and fibrosis in a paracrine manner. The IL-1 receptor antagonist (IL1RN) secreted by MSCs appears to be a competitive inhibitor of IL-1α and IL-1β, blocking IL-1 and TNF-α signaling in lung tissues [9, 29]. MSCs also promote the expression of the anti-inflammatory factor IL-10, which suppresses the activity of macrophages, neutrophils, and DCs and inhibits Th1 response and the secretion of DC inflammatory cytokines [5]. Nuclear factor (NF)-κB signaling plays an important role in inflammation-related diseases. NF-κB activation enhances the secretion of the chemokine (C-X-C motif) ligand (CXCL) 8 and 11, which induces the production of neutrophils, and it enhances the secretion of inflammatory cytokines and proteolytic enzymes by Th1 cells [30]. Studies have shown [31] that human bone marrow MSCs can block the activation of NF-κB, thereby reducing the occurrence of inflammation [32, 33].

TGF-β1 is considered to play a crucial role in RILI, particularly in RILF [3, 34]. An acute and long-lasting increase in TGF-β1 expression was observed in mouse lungs, following 12 Gy of thoracic irradiation [34–36]. The predominant localization of TGF-β1 was in areas of inflammatory cell infiltrates and fibrosis, suggesting the involvement of this cytokine in the pathogenesis of RILF. During wound healing, TGF-β1 enhances IL-1 production in monocytes, which in turn exerts a mitogenic effect on fibroblasts. Therefore, inhibition of TGF-β1 signals may reduce RILF [35, 37]. Studies have shown that MSCs downregulate TGF-β1 levels by secreting PGE2 and the hematopoietic growth factor (HGF) [38]. PGE2 inhibits TGF-β1-induced proliferation of fibroblasts and induces myofibroblast apoptosis by increasing the phosphatase and tensin homolog protein activity. In addition, HGF can reduce EMT, thereby inducing myofibroblast apoptosis.

Based on their natural features, genetically modified MSCs have received considerable attention as a preventive measure against RILI. Chen et al. used MSCs harboring genetically modified superoxide dismutase (SOD) for treating RILI, which showed improved anti-inflammatory and anti-fibrotic properties [39]. Furthermore, Wei et al. investigated whether injection of human umbilical cord-derived MSCs (UC-MSCs) overexpressing SOD3 at the established fibrosis stage exerted beneficial effects in a mouse model of RILF [40]. They observed that early treatment with UC-MSCs alone significantly reduced RILF in mice via paracrine signaling, an outcome that was further improved upon administration of SOD3-overexpressing UC-MSCs. These results suggest that SOD3-overexpressing UC-MSCs may be used as a cell-based gene therapy for treating RILF.

#### Resistance to oxidative stress

ROS generated after lung radiation [6] can directly damage proteins and generate hydroxyl free radicals that will in turn cause DNA damage [7, 8]. Moreover, ROS can also damage mitochondrial DNA and induce inflammation and immune responses [41, 42], as well as cell loss and increase vascular permeability, protein exudation, and apoptosis of alveolar I-type epithelial cells [6]. However, MSCs possess antioxidant properties and can produce potent antioxidant enzymes such as SOD1, SOD3, and MnSOD [43, 44]. SOD1 catalyzes the conversion of superoxide radical into oxygen and hydrogen peroxide, thereby protecting lungs from radiation-induced endothelial damage [43]. Similarly, MSCs harboring engineered *SOD3* or *MnSOD* genes show better anti-fibrotic effects in RILI than non-modified MSCs [39]. Therefore, the positive effect of SOD is probably due to its ability to catalyze the dismutation of the superoxide radical into oxygen and hydrogen peroxide, thereby protecting injured cells from ROS generated in RILI [45]. Interestingly, Li et al. [44] further demonstrated that the antioxidant ability of MSCs in the hypoxic environment increased significantly due to upregulation of indoleamine-2,3-deoxygenase (IDO). Studies have shown that MSCs exhibit resistance to radiation in low-oxygen environments [46, 47] and also harbor the DNA double-strand break repair system [48–50], which gives them a natural protection to survive within the RILI microenvironment.

#### Inhibition of EMT

Radiation damages AECs and endothelial cells and triggers the release of pro-inflammatory cytokines, which promote the activation of fibroblasts and induce EMT in AECs, ultimately promoting the development of RILF [3, 9, 51]. The Wnt/β-catenin signaling pathway plays a crucial role in inducing EMT [52]. Increasing evidences suggests that AECs undergoing EMT respond to TGF-β1 and Wnt signaling pathways [53, 54]. Zhang et al. [53] found that co-culture of normal human lung fibroblasts with umbilical cord-derived MSCs weakened the activation of the Wnt/β-catenin signaling, thereby suppressing the differentiation and proliferation of fibroblasts [52]. Therefore, the use of UC-MSC therapy can be beneficial for alleviating lung fibrosis. In addition, Dong et al. observed that human adipose tissue-derived MSCs can protect type II AECs from radiation-induced EMT by secreting HGF [34].

#### Immunosuppressive effects of MSCs

RILI can lead to the activation of the immune system. Recruitment of immune cells and the subsequent cascade of cytokine production result in various degrees of lung inflammation after radiation. However, MSCs can regulate the proliferation, activation, and effector functions of T lymphocytes, DCs, and macrophages in the pathogenesis of inflammatory lung diseases [55].

MSCs can inhibit CD4^+^ helper and CD8^+^ cytotoxic T lymphocytes via cell-to-cell contact and paracrine mechanisms [56]. Interestingly, the immunosuppressive mechanisms mediated by MSCs of different species vary [55, 57]. Human- or monkey-derived MSCs can inhibit T cells via IDO, whereas mice-derived MSCs inhibit the proliferation of T cells via nitric oxide [57]. IDO degrades tryptophan into kynurenine and toxic metabolites that will inhibit the T cell proliferation. Glennie and co-workers demonstrated that MSCs can induce the inactivation of activated T cells [58]. Furthermore, TGF-β1, PGE2, and heme oxygenase-1 secreted by MSCs can inhibit the proliferation of T cells [59, 60]. MSCs can attenuate the production of IL-2, which is involved in the activation of the JAK/STAT pathway in T cells, thereby inhibiting the proliferation of activated T cells [61]. Heme oxygenase-1 suppresses T cell proliferation and IL-2 secretion by inhibiting the ERK/MAP kinase pathway. Thus, upregulation of heme oxygenase-1 also contributes to T cell suppression [3]. In addition, lactose-1 and signal protein-3A, which are highly expressed by MSCs, are soluble factors that inhibit T cell proliferation [62]. More importantly, the inhibitory effect of TNF-α-stimulated MSCs on T cells is enhanced, which indicates that the level of inflammatory mediators has an important effect on the immunosuppressive function of MSCs [63].

MSCs can also regulate DC maturation. MSCs downregulate HLA II, CD80, CD86, and IL-12 in DCs, resulting in the inhibition of DC maturation [64]. These immature DCs can disable Th1 cells. Furthermore, MSCs can induce mature DCs to differentiate into tolerant DCs, increasing the production of anti-inflammatory cytokines and decreasing the production of inflammatory factors. These effects could be further enhanced by the overexpression of HGF in MSCs [65].

In addition, MSCs can promote the differentiation of macrophages from the classic M1 inflammatory phenotype into the anti-inflammatory M2 phenotype by secreting PGE2, TNF-α, TNF-α-stimulated gene/protein 6 (TSG-6), IL-6, and IDO [66]. The PGE2 released by MSCs bind to its receptors, EP2 and EP4, on macrophages and activates downstream pathways that will polarize macrophages into the M2 phenotype [66]. In the experimental model of zymosan-induced peritonitis, MSCs secreted TSG-6 that interacts with CD44 on macrophages to reduce the Toll-like receptor 2 (TLR2)/NF-κB signal, thereby reducing the secretion of pro-inflammatory mediators [67]. Meliv and colleagues found that MSCs co-cultured with monocytes secrete a high level of IL-6, which promotes differentiation of monocyte and consequent production of the anti-inflammatory IL-10 [65]. The neutralization of IL-6 reversed this inhibitory effect of MSCs [65]. In an inflammatory environment, IDO secreted by MSCs catalyzes the degradation of tryptophan to kynurenine, which can directly inhibit T cell proliferation via cell-to-cell contact with monocyte-differentiated M2 macrophages [68]. In addition, the polarization of macrophages induced by MSCs promotes the generation and expansion of regulatory T cells [55]. Considering the immunosuppressive effect of MSCs, we speculated that MSCs can also reduce RP and RILF by releasing immunosuppressive factors, and thereby exert therapeutic effects for the treatment of RILI.

#### Release of extracellular vesicles (EV)

MSCs also release large numbers of EVs, which mediate tissue repair and anti-inflammatory effects during lung pathogenesis. The paracrine effect of EVs has also been extensively investigated [69]. EVs are of three types: exosomes, microvesicles, and apoptotic bodies [9, 70]. Studies have shown that MSC-exosomes can downregulate the level of inflammatory factors and upregulate the level of IL-10 [71]. Interestingly, the miRNA profile of patients undergoing radiotherapy is altered, which suggests that miRNAs may participate in RILI. Li et al. [71] reported that MSC-exosomes shuttle the miRNA-181c, which can downregulate IL-1β and TNF-α levels and upregulate IL-10. Furthermore, MSC-exosome-shuttled miRNAs can inhibit inflammation and fibrosis via cell regeneration and immunoregulation [72]. Reports have also shown that miRNAs shuttled by MSCs play a major role in preventing inflammation and fibrosis, which further supports the potential therapeutic function of miRNAs released by MSCs for RILI [73]. In summary, MSC-EVs represent a promising therapy tool for RILI.

### Problems associated with MSC-based therapy for RILI

In recent years, numerous animal models of RILI were used to test the therapeutic effect of MSCs, which were shown to be effective in alleviating RILI manifestations [74, 75]. However, clinical research is still lacking. The tumor-promoting effect of MSCs limits their translational application into the clinic for patients with RILI. In addition, the lack of comparative characterization of murine and human MSCs may also limit the direct translation into clinical trials of the findings based on preclinical animal models. Thus, the limitations associated with the tumorigenic potential of MSCs in clinical trials should also be considered. Furthermore, studies should focus more clearly on identifying factors responsible for the therapeutic effects of MSCs.

Certain factors affect the therapeutic efficacy of MSCs. The time window for administering therapies plays an important role in RILI treatment [75]. Furthermore, the therapeutic effects of different sources and doses of MSCs may vary [27, 76]. Wang et al. reported that UC-MSCs decreased the inflammatory response [76], whereas Jiang et al. observed that adipose tissue-derived MSCs not only regulated the anti-inflammatory factors, but also anti-fibrotic factors during RILI treatment [77]. Other studies have reported that the therapeutic effects of MSCs are limited, and most treatments still depend on the paracrine mode of action of MSCs [3, 78]. Although an increasing number of studies have indicated the therapeutic potential of MSCs, the safety of MSC therapy remains debatable [74]. Hence, MSC-based therapy for RILI still has a long way to go before it can be used in the clinic.

## Conclusions

RILI is a serious and complicated lung disease lacking specific treatments. Recently, several studies suggested MSC-based therapy as a promising treatment for RILI. However, further studies are warranted to address the safety, optimal dose, and time window of MSC treatment. Nowadays, genetically modified MSCs have attracted the attention of many experts in the field and may represent the next for the development new therapeutic strategies. In conclusion, MSC therapy has great potential for managing RILI.

## Data Availability

Not applicable.

## Abbreviations

RILI: Radiation-induced lung injury
MSCs: Mesenchymal stem cells
RP: Radiation pneumonitis
RILF: Radiation-induced lung fibrosis
ROS: Reactive oxygen species
IFN-γ: Interferon γ
IL-4: Interleukin 4
IL-2: Interleukin 2
IL-13: Interleukin 13
TGF-β1: Transforming growth factor-β1
PGE2: Prostaglandin E2
IL-10: Interleukin 10
RAAS: Renin-angiotensin-aldosterone system
IL-1: Interleukin 1
IL-6: Interleukin6
TNF-α: Tumor necrosis factor α
EMT: Epithelial mesenchymal transformation
MMP2: Matrix metallo proteinase-2
MMP9: Matrix metallo proteinase-9
SOD1: Superoxide dismutase 1
SOD3: Superoxide dismutase 3
MnSOD: Manganese superoxide dismutase
IL1RN: Interleukin-1 receptor antagonist
HGF: Hematopoietic growth factor
EVs: Extracellular vesicles
IL-8: Interleukin 8
TSG-6: TNF-α stimulated gene/protein 6
IDO: Indoleamine 2,3-dioxygenase
UC-MSCs: Umbilical cord-derived mesenchymal stem cells
